# Supplementary material for: A Novel Methylene Blue Indicator-Based Aptasensor for Rapid Detection of Pseudomonas aeruginosa
Source: Int J Mol Sci. 2024 Oct 30;25(21):11682. doi: 10.3390/ijms252111682 (PMC11547117; doi:10.3390/ijms252111682)
Supplement: Supplementary file 1 [file ijms-25-11682-s001.zip › ijms-3274466-supplementary.pdf]

Supplementary materials

A novel methylene blue indicator based aptasensor for rapid detection of *Pseudomonas aeruginosa*

Somayeh Maghsoomi [1,2], Julia Walochnik[2], Martin Brandl[1], Mai-Lan Pham[1*]

[1] Center for Water and Environmental Sensors, Department for Integrated Sensor Systems, University for Continuing Education Krems, Dr.-Karl-Dorrek-Straße 30, 3500 Krems an der Donau, Austria

[2] Institute of Specific Prophylaxis and Tropical medicine, Medical University of Vienna, Kinderspitalgasse 15, 1090 Vienna, Austria.

*C**orresponding author**: mai.pham@donau-uni.ac.at

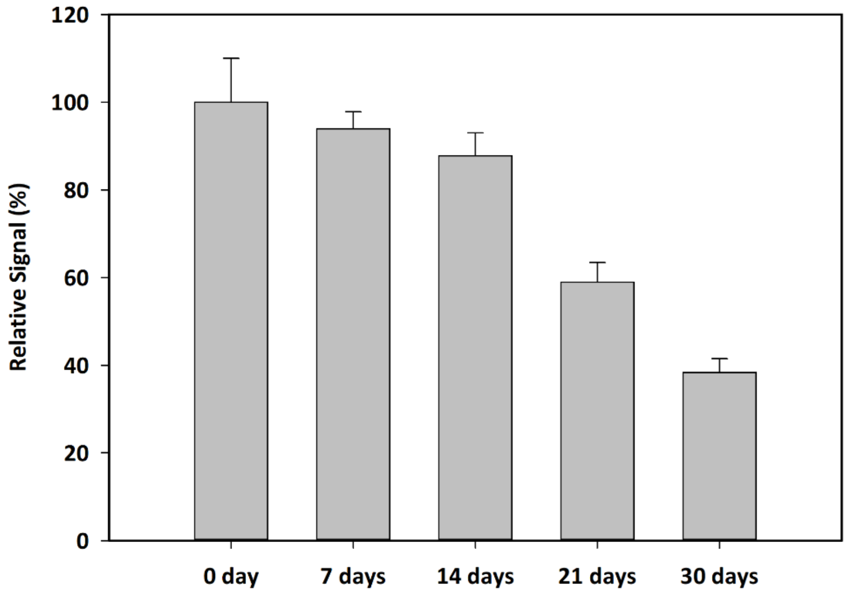

Figure S1: Stability of the aptasensor stored at 4 °C in Tris buffer, pH 7.0. All meas-urements were performed on three different modified SPEs by SWV in Tris buffer (pH 6.0) containing 50 mM MB. The signal obtained from the first day of storage is considered as 100 %.

Table S1: Current peaks measured by SWV in the Tris buffer supplemented with or without methylene blue (MB) of aptasensors incubated with or without interfering bacteria. Each measurement was conducted triplicate in three different aptasensors. The results are expressed as the mean ± standard deviation (SD).

	**Current peak (µA)**	
	**Without MB**	**With MB**
**Absence of bacteria**	188.433 ± 5.655	387.737 ± 9.991
P. aeruginosa	54.502 ± 0.821	107.305 ± 2.642
Bacterial mixture*	51.814 ± 0.000	99.673 ± 1.354
E. coli	14.902 ± 0.228	29.073 ± 0.294
E. faecalis	16.432 ± 0.493	30.258 ± 10.363
S. aureus	10.410 ± 0.156	25.186 ± 0.513
P. putida	11.993 ± 0.015	26.173 ± 0.075

* The aptasensor was incubated with a mixture of tested interfering bacterial strains including P. aeruginosa.
